# BCL6 (B-cell lymphoma 6) expression in adenomyosis, leiomyomas and normal myometrium

**DOI:** 10.1371/journal.pone.0317136

**Published:** 2025-02-04

**Authors:** Loreta Canivilo Salas, Bruna Mielczarski, Raquel Camara Rivero, João Sabino Lahogue da Cunha Filho, Ricardo Francalacci Savaris

**Affiliations:** 1 Postgraduate Program in Medicine, Surgical Sciences, Universidade Federal do Rio Grande do Sul, Porto Alegre, Rio Grande do Sul, Brazil; 2 Department of Obstetrics and Gynecology, Universidade Federal do Rio Grande do Sul, Porto Alegre, Rio Grande do Sul, Brazil; Teikyo University, School of Medicine, JAPAN

## Abstract

Adenomyosis and leiomyomas are common benign uterine disorders characterized by abnormal cellular proliferation. The BCL6 protein, a transcriptional repressor implicated in cell proliferation and oncogenesis, has been linked to the pathogenesis of endometriosis. This study investigates BCL6 expression in adenomyosis, leiomyomas, and normal myometrium using immunohistochemistry and deep learning neural networks. We analyzed paraffin blocks from total hysterectomies performed between 2009 and 2017, confirming diagnoses through pathological review. Immunohistochemistry was conducted using an automated system, and BCL6 expression was quantified using Fiji-ImageJ software. A supervised deep learning neural network was employed to classify samples based on DAB staining. Our results show that BCL6 expression is significantly higher in leiomyomas compared to adenomyosis and normal myometrium. No significant difference in BCL6 expression was observed between adenomyosis and controls. The deep learning neural network accurately classified samples with a high degree of precision, supporting the immunohistochemical findings. These findings suggest that BCL6 plays a role in the pathogenesis of leiomyomas, potentially contributing to abnormal smooth muscle cell proliferation. The study highlights the utility of automated immunohistochemistry and deep learning techniques in quantifying protein expression and classifying uterine pathologies. Future studies should investigate the expression of BCL6 in adenomyosis and endometriosis to further elucidate its role in uterine disorders.

## Introduction

Adenomyosis is a benign uterine disease characterized by the pathological presence of endometrial glands and stroma within the myometrium [1]. Uterine adenomyosis affects women between the ages of 16 and 60, with a prevalence of around 0.8% [2] but can reach up to 80% among premenopausal women with infertility and chronic pelvic pain [3]. This condition causes chronic pelvic pain, dysmenorrhea, dyspareunia, infertility, and adverse obstetric outcomes [4]. Microscopically, adenomyosis exhibits ectopic endometrial glands and stroma surrounded by hypertrophic myometrium [5, 6]. It is thought to result from an invagination of the endometrium following the disruption of the junctional zone between the basal endometrium and the myometrium [7–9]. Molecular validation by Inoue et al. confirmed that adenomyosis develops from eutopic endometrium through the identification of somatic mutations using next-generation sequencing [10].

There is a possibility that adenomyosis and endometriosis represent different phenotypes of the same disease [11]. For instance, the same somatic mutations in the KRAS gene mutation can be detected in both adenomyosis and the corresponding eutopic endometrium, suggesting a clonal relationship [3]. The KRAS gene provides instructions for the production of a protein called K-Ras, which is part of the RAS/MAPK signaling pathway involved in cell proliferation and differentiation. Mutations in KRAS stimulate pathways that increase cell survival and proliferation and are associated with progesterone resistance in adenomyosis and endometriosis [12]. KRAS activation in cells with positive progesterone receptors stimulates the expression of the BCL6 protein, which is implicated in the pathogenesis of endometriosis [13]. The KRAS gene belongs to a class of genes known as oncogenes, as its mutation is associated with cancer [14].

BCL6 (B-cell lymphoma 6) is a transcriptional gene repressor, which is necessary for the development of B cells and oncogenesis [15]. It is associated with increased cell proliferation through the repression of genes such as p53 [16]. Our group reported that BCL6 is highly overexpressed in the endometrium of women with endometriosis during the secretory phase of the menstrual cycle compared to women without endometriosis [17]. Given the similarities between endometriosis and adenomyosis and the increased expression of KRAS in both conditions [9], it is hypothesized that the BCL-6 protein may also be increased in adenomyosis.

Considering the shared molecular pathways and genetic mutations, related to proliferation, observed in adenomyosis and endometriosis, it is plausible to extend the investigation to other uterine pathologies, such as leiomyomas. Leiomyomas, or uterine fibroids, are benign smooth muscle tumors of the uterus that also exhibit aberrant cellular proliferation and differentiation [18]. Although there is limited evidence linking BCL6 expression to leiomyomas, some authors have shown higher mRNA expression of BCL6 in leiomyomas, compared to normal myometrium [19]. In contrast, one study reported BCL6 protein overexpression in only one out of nine leiomyoma samples using a subjective scoring system [20].

Given this context and the implication of BCL6 in cell proliferation, the objective of this study is to verify BCL6 expression in cases of adenomyosis, leiomyomas, and controls (normal myometrium and endometrial glands). We will use an automated immunohistochemistry machine and computer-based image analysis for the quantification and classification of BCL6 expression in these groups. We hypothesize that BCL6 may also play a role in the pathogenesis of leiomyomas and adenomyosis.

## Material and methods

### Ethics statement

This study was submitted and approved by Hospital de Clínicas de Porto Alegre Ethical Review Board, under the approval number 2022/0177 and registered at *Plataforma Brasil* under the certificate of submission for ethical analysis (CAAE 58868422.6.0000.5327).

### Study design and setting

In this case-control study, paraffin blocks were obtained from the pathological archive of total hysterectomies performed at Hospital de Clínicas in Porto Alegre, Brazil. dated between January 1st, 2009, and December 30th, 2017. The original slides and their pathological report were reviewed by a board-certified pathologist to confirm the diagnosis of fibromyomas and normal myometrium. Sample collection was conducted between September 20, 2022, and April 30, 2023, and the analysis of the photomicrographs and statistical analysis were performed until April 30, 2024.

### Patients and methods

Women with diagnosis of abnormal uterine bleeding and also underwent a total hysterectomy for benign conditions (e.g., uterine prolapse, fibroleiomyoma, adenomyosis) were eligible and included in the sample. Patients with malignant conditions were excluded.

### Variables and outcome

BCL6 protein expression was the primary continuous variable, i.e., DAB units, varying between 0 (no expression) and 255 units (highest expression), and it was the primary measured outcome.

Normal myometrium and glands (control group), adenomyosis and leiomyomas were categorical data. Other variables were age (years-old) and ethnicity (white and non-white), weight (kg), and parity.

### Data sources / measurement

#### Immunohistochemistry

Immunohistochemistry methodology was performed using an automated technique with the BenchMark ULTRA IHC/ISH system (Roche diagnostics, Rotreuz, Switzerland). Primary antibody against BCL6 was GI191E.A8 Cell Marque. Immunostaining was performed with 3, 3’-diaminobenzidine (DAB). Human lung cancer samples were used as external positive control. Details of the automated setup is in the supplement (see S1 File).

#### Photomicrographs

Images from stained sections were obtained using an optical microscope (Olympus BX51 microscope; Olympus Optical Co., Tokyo, Japan) with a 10x objective PlanC N (numerical aperture 0.25 mm, Olympus). A digital color camera (Olympus DP73; OM Digital Solutions Co., Tokyo, Japan) captured digital images, at a size of 4800 x 3600 pixels (resolution: 1 mm = 4130 pixels), under standard conditions for Fiji-ImageJ analysis. In adenomyosis cases, the whole tissue section with adenomyosis was photographed, while in leiomyoma and normal cases, three pictures were taken in each group.

#### Image analysis with Fiji-ImageJ

Photomicrographs were coded and blindly analyzed using Digital HSCORE (D-HSCORE) as previously reported [21–23], using Fiji for ImageJ [24]. Briefly, the selection of the regions of interest (ROI) in controls were glands and myometrium; in adenomyosis, the glands, and in the leiomyoma group, the area with leiomyoma. After selecting the ROI, images were submitted for “color deconvolution” analysis. The image with DAB staining was used for analysis. A VBA script was used for automating the analysis (see supplement for S1 Fig).

#### Supervised deep learning neural network

The 120 photomicrographs of DAB-only images, derived from the original set and without ROI selection, were submitted to supervised deep learning neural network (SDLNN) analysis using Orange 3.31.0 software (University of Ljubljana, Slovenia) as previously reported [25].

#### Bias

We tried to reduce bias by using D-HSCORE and SDLNN.

#### Study size

The sample size for ImageJ analysis was based on the existing literature [26] to ensure a statistical power of 95%, an α error of 5%, and a standard deviation of 6 arbitrary units of DAB. This was necessary to detect an increase from a baseline of 30 DAB units (control) to 40 DAB units in conditions such as leiomyomas or adenomyosis. With these parameters, a minimum of 10 samples per group was required. Conversely, the sample size for the supervised neural network analysis was selected based on the availability of photomicrographs from the slides.

#### Quantitative variables

The average DAB units intensity, derived from up to three images obtained, as previously described [22].

### Statistical methods

Categorical data across groups were analyzed using the chi-squared test for trend. For continuous data concerning BCL6 expression in arbitrary DAB units, comparisons between groups were conducted using the Kruskal-Wallis test accompanied by Dunn’s multiple comparisons test, or ANOVA with Welch’s correction when there were differences in standard deviations among groups. The BCL6 expression in normal myometrium was compared to that in leiomyoma using the Student’s t-test with Welch’s correction. D’Agostino & Pearson omnibus normality test was used to verify the Gaussian distribution. GraphPad Prism version 10.2.2 for Macintosh (GraphPad Software Inc. San Diego, CA) was used for statistical analysis.

Orange 3.31.0 software (University of Ljubljana, Slovenia) was used for deep neural network image embedding recognition model analysis used Adam algorithm [27] for optimization using the same parameters as previously reported [25].

## Results and discussion

### Participants and descriptive data

The study collected a total of 39 samples, consisting of 14 from normal endometrial glands, 12 from adenomyosis, and 13 from fibromyomas. No significant differences were found among these groups, as shown in Table 1.

**Table 1:** Characteristics of the studied population.

Characteristics	Control (n = 14)	adenomyosis (n = 12)	fibromyoma (n = 13)	*p*
Age (years) median (range)	55.5 (37–93)	39 (39–51)	46 (30–93)	0.3^a^
Ethnicity n(%)				
White	12(85.7)	11(91.7)	12(92.3)	0.9^b^
non-white	2(8.3)	1(8.3)	1(7.6)	
Weight (kg) mean (SD); n	76.7 (18.4); 6	72.1 (11.7); 8	72.6(9.5); 6	0.7^c^
Parity—mean (SD); n	3.1 (1.7); 6	3.3 (2.5); 9	2.3 (1.5); 8	0.5^c^

^a^ Kruskal-Wallis

^b^ Chi-squared for trend

^c^ Welch’s ANOVA

SD = standard deviation.

Some data was not available in patients’ electronic records. Available numbers are mentioned in the table.

### Outcome data and main results

**Fig 1:**
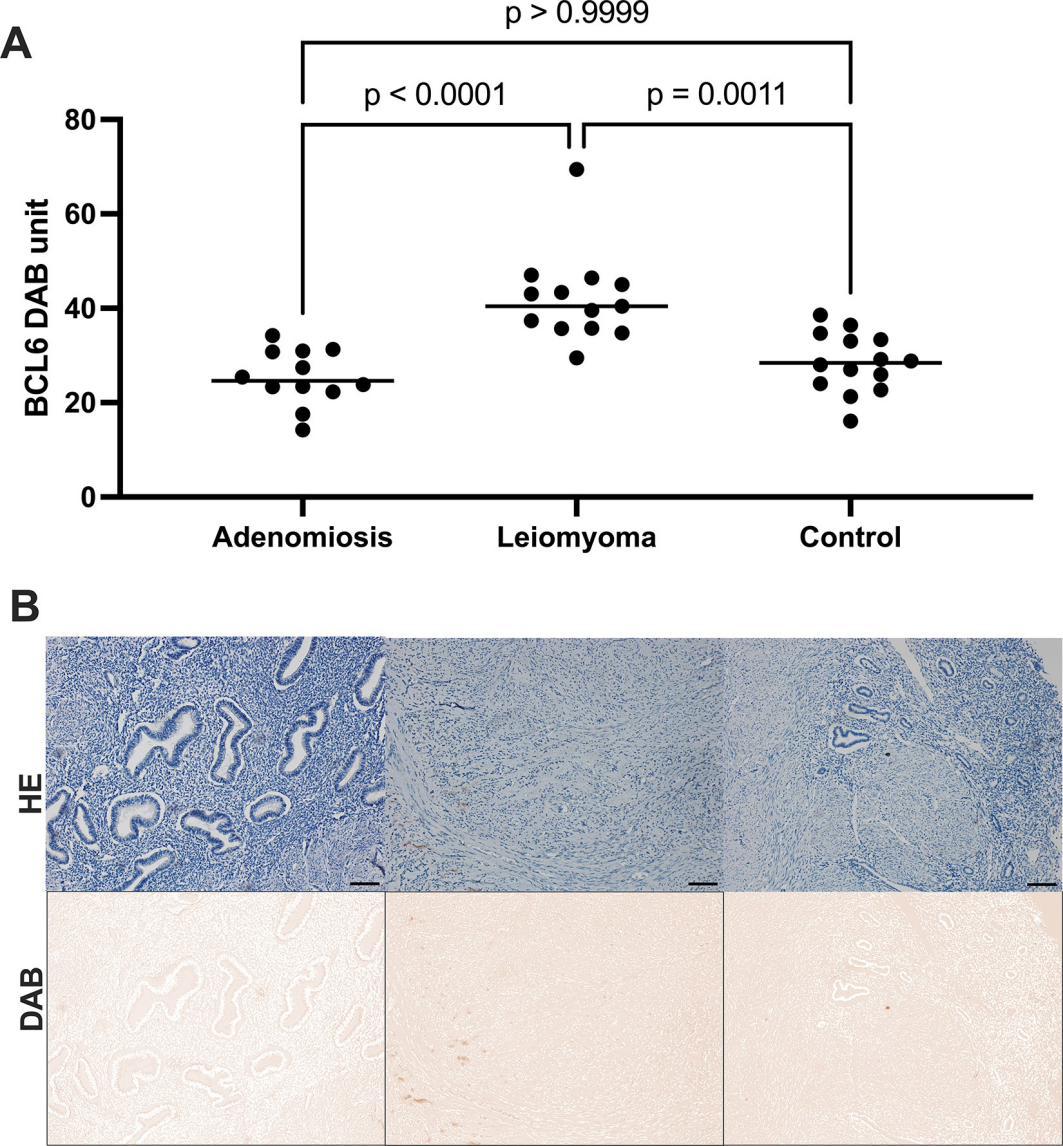
A scatter dot plot of median 3, 3’-diaminobenzidine expression (DAB) of BCL6 in cases with adenomyosis, leiomyomas and controls. A significant difference between controls and leiomyoma, and between adenomyosis and leiomyoma were identified (Kruskal-Wallis with Dunn’s post-hoc test). Each dot represents a sample, bars represent median values.

The median BCL6 protein expression in DAB units was significantly different among groups (*p*<0.0001, Kruskal-Wallis test). The BCL6 expression (median; range) was higher in leiomyomas (40.4; 29.5 to 69.4), compared to either adenomyosis (24.6;14.2 to 34.2) or controls (28.4;16 to 38.5). However, no difference was found between controls and adenomyosis (Fig 1).

Photomicrographs of BCL6 protein expression in (A) hematoxylin+DAB (HE+DAB) staining in adenomiosis, leiomyomas and control, and in (B) respective DAB expression of the respective photomicrographs in A after color deconvolution. Bar represents 100 μm.

**Fig 2:**
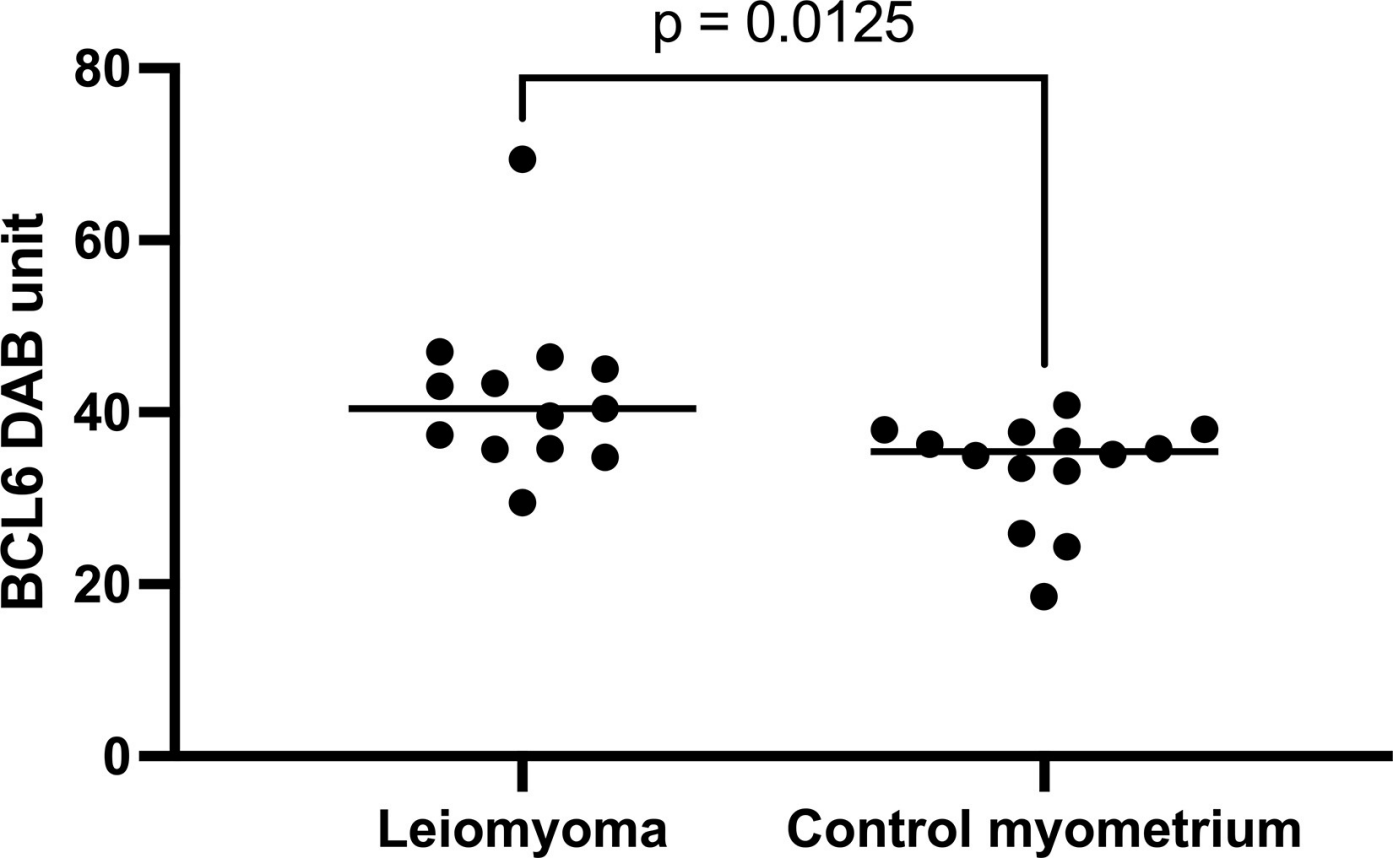
A scatter dot plot of median 3, 3’-diaminobenzidine expression (DAB) of BCL6 in cases with leiomyomas and myometrium from controls. A significant difference between controls and leiomyoma was identified (Mann-Whitney test). Each dot represents a sample, bars represent median values.

The comparison between leiomyoma and normal myometrium was statistically significant (*p* = 0.0125, Mann-Whitney test): control myometrium (35.43; 18.59 to 40.81), leiomyoma (40.41; 29.53 to 69.45) as shown in Fig 2.

The area (mm^2^) analyzed among groups was not significantly different (values are median; range): adenomyosis (0.07; 0.005 to 0.36), leiomyomas (0.08; 0.01 to 0.29) and control (0.15; 0.07 to 20); *p* = 0.9; Kruskal-Wallis.

The raw data from image analyses are in the supplement (see S1 Dataset).

### Supervised deep learning neural network analysis

**Fig 3:**
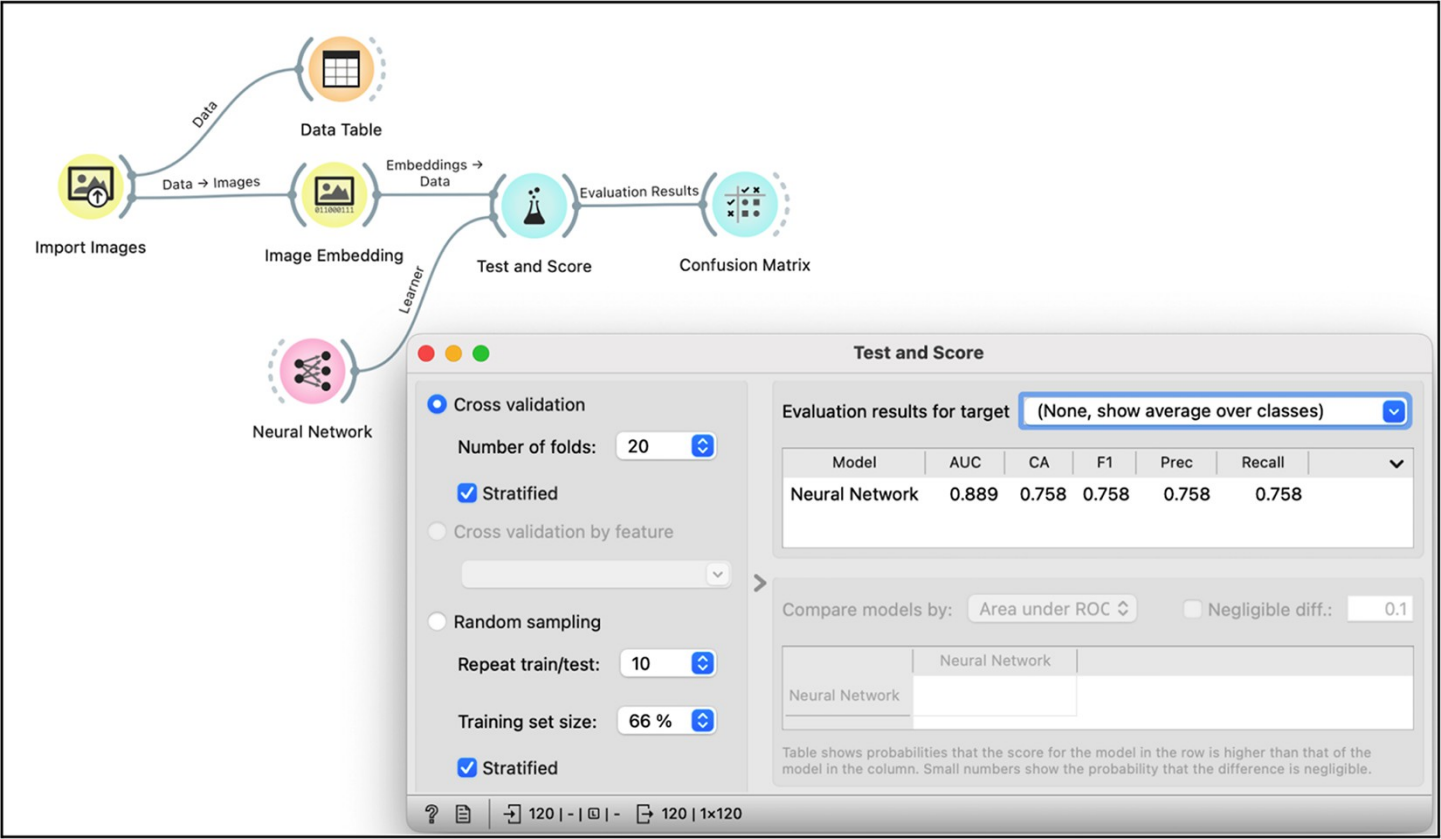
Flowchart of the supervised neural network analysis using Orange 3 with test and score results. AUC: area under the curve; CA: classification accuracy; F1: weighted harmonic mean of precision and recall; Prec: the proportion of true positives among instances classified as positives; Recall: the proportion of true positives among all positives instances in the data.

The SDLNN analysis yielded an area under the curve of 88.9%, using a total of 120 photomicrographs with DAB staining only, derived from Fiji-ImageJ (Fig 3). Further details are depicted in Table 2.

**Table 2:** Performance of supervised neural network analysis for classifying samples as adenomyosis (Adeno), leiomyoma (Leio) and control based on DAB expression only.

confusion matrix					Parameter	Results	95%CI
		predicted					
		Adeno	Leio	Control	Accuracy	75.8	(67.4–82.6)
actual	Adeno	27	1	11	F1	75.8	
	Leio	1	36	2	Precision	75.8	(67.4–82.6)
	Control	12	2	28	Recall	75.8	(67.4–82.6)

F1: weighted harmonic mean of precision and recall

Precision: the proportion of true positives among instances classified as positive

Recall: the proportion of true positives among all positive instances in the data

The findings of this study reveal that BCL6 expression is higher in leiomyomas, compared to normal myometrium, but it is not significantly different between adenomyosis and controls.

These results are consistent with some aspects of the existing literature while diverging from others, highlighting the complex role of BCL6 in the pathophysiology of uterine disorders. BCL6 is one of the human proto-oncogenes and it is associated with increased cell proliferation through the repression of genes such as p53 [16]. Most studies investigating leiomyomas focus on microarray expression compared to normal myometrium [19, 28–30]. An *in silico* analysis of BCL6 mRNA expression, using the whole-genome expression, reveals that BCL6 mRNA expression is elevated in leiomyomas, compared to normal myometrium [19]. From the perspective of BCL6 protein expression, the study by Walters et al. is the only one that has examined BCL6 protein expression in leiomyomas [20]. Contrary to our results, Walters et al found only one positive case, out of nine leiomyomas (11.1%; 95% CI: 1.9% to 43.5%), with nuclear BCL6 positivity. Several factors could explain these differences, including variations in the BCL6 antibodies used, the methodologies employed, and the approaches to immunoexpression analysis. In our study, we utilized the GI191E.A8 antibody from Roche and performed a computer-based analysis to compare BCL6 expression in leiomyomas with normal myometrium. In contrast, Walters et al. used the PG-B6P antibody from Dako and relied on a visual scoring method, categorizing the expression as negative, 1+, 2+, or 3+ [20]. These factors may explain the divergent results. Our group has shown that different antibodies against the same protein, such as Mucin 1, may yield different results [31]. Additionally, automated methods have been shown to be reliable and superior to manual scoring [32]. In leiomyomas, the overexpression of BCL6 might contribute to the abnormal proliferation of smooth muscle cells, leading to tumor growth.

Regarding adenomyosis, our findings that BCL6 expression levels are not significantly different compared to controls aligns with some previous studies. A GEO analysis of the data from a study by Juárez-Barber et al. (GSE244236) revealed that no significant difference in gene expression exists between adenomyosis and normal endometrial glands [33]. The lack of significant BCL6 overexpression in adenomyosis compared to endometrial glands from controls, found herein, supports the notion that BCL6 may not play a major role specifically in the pathogenesis of adenomyosis.

Our data is unique and needs to be confirmed. We acknowledge that different antibodies, immunohistochemical analysis and technique may provide different results. However, we provided detailed information about our methodology for replication.

This study has some limitations. We did not analyze subgroups, such as focal versus diffuse adenomyosis or leiomyoma locations, nor did we conduct mechanistic experiments. The lack of difference between controls and adenomyosis could be related to insufficient statistical power. A post-hoc analysis revealed that we had only 25.6% power to confirm the absence of a difference between these two groups. To identify a significant increase in the adenomyosis group from 25.4 to 28.52 DAB units compared to the control group, it would be necessary to increase our sample size to 106 samples in each group, maintaining the same power of 95% (1—beta error of 5%).

The results here are strengthened by several aspects. The use of Fiji-ImageJ software and the use of VBA script allows others to reproduce our data and reduces the subjective bias of DAB quantification. The SDLNN was able to classify 91 out of 120 photomicrographs based only on DAB expression, confirming the results obtained with Fiji-ImageJ analysis. This technique has been used previously by our group and it seems promising [25]. The criteria used for classification of the slides by the artificial intelligence was not completely understood; it is likely that the artificial intelligence used other factors beyond DAB expression, for instance, the presence of glands, to classify the photomicrographs. Of note, a higher degree of classification was observed in the leiomyoma group. The use of an automated immunohistochemistry protocol, commonly used in the laboratory practice and the negative controls are good laboratory practice.

In conclusion, we were not able to verify a difference between BCL6 expression between adenomyosis and normal endometrial glands, however, a higher and significant expression was observed in leiomyomas. Further studies may investigate the expression of BCL6, using our methodology, between adenomyosis and endometriosis.

## Supporting information

S1 File Immunohistochemical technique and settings used in BenchMark ultra. (DOCX)

S1 Fig Script in VBA (Visual Basic for Applications) used in Fiji ImageJ to process H-DAB. In line 31 saveAs(“Tiff”, “<put here the path where the files should be saved>” + name).
(TIFF)

S2 Fig Script to analyze DAB images into ROI. A video of the process can be found at https://www.youtube.com/watch?v=9nLRSquNa5Q.
(TIFF)

S1 Dataset Raw data of image analyses. (XLSX)
