# Supplementary material for: BCL6 (B-cell lymphoma 6) expression in adenomyosis, leiomyomas and normal myometrium
Source: PLoS One. 2025 Feb 4;20(2):e0317136. doi: 10.1371/journal.pone.0317136 (PMC11793761; doi:10.1371/journal.pone.0317136)
Supplement: S1 File — (DOCX) [file pone.0317136.s001.docx]

**Supplement**

# **Immunohistochemical technique and settings used in BenchMark Ultra**

Samples were submitted to standard immunohistochemical staining using BenchMark Ultra IHC/ISH , according to U ultraView DAB protocol (v1.02.0018) for BCL6 (GI191E.A8) Cell Marque.

Procedure

1. Activate Mixers
2. Heat slide to [75 C], and incubate for [4 minutes] (Heating)
3. Deactivate Mixers
4. [Set temperature to 72°C]
5. Heat Slide to [72° C) from Medium Temperatures (Deparaffination)
6. Incubate for 4 minutes
7. Apply EZPrep Volume Adjust
8. Wash Slide with EZ Prep
9. Apply EZPren Volume Adjust
10. Apply Coverslip
11. Wash Slide with EZ Prep
12. Apply EZPrep Volume Adjust
13. Apply Coverslip
14. Wash Slide with EZ Prep
15. Apply Depar Volume Adjust
16. Apply Coverslip
17. Activate Mixers
18. Deactivate the slide heater
19. Pause Point (Pause Zone)
20. Brief- [8 Minutes of Conditioning]
21. Wash Slide with EZ Prep
22. Apply Cell Conditioner #1 prolonged
23. Apply CC Coverslip prolonged
24. [Set temperature to 95°C]
25. Heat slide to [95°C], and incubate for 8 minutes (Cell Conditioner #1)
26. Apply Cell Conditioner #1
27. Apply CC intermediate Coverslip without BB (barcode blowoff)
28. Apply CC intermediate Coverslip without BB (barcode blowoff)
29. Apply Cell Conditioner #1
30. Apply CC intermediate Coverslip without BB (barcode blowoff) [SOFT]
31. [SOFT]
32. Apply Cell Conditioner #1
33. Apply CC intermediate Coverslip without BB (barcode blowoff)
34. Apply Cell Conditioner #1
35. Apply CC intermediate Coverslip without BB (barcode blowoff)
36. Apply Cell Conditioner #1
37. Apply CC intermediate Coverslip without BB (barcode blowoff)
38. Apply Cell Conditioner #1
39. Apply CC intermediate Coverslip without BB (barcode blowoff)
40. Apply Cell Conditioner #1
41. Apply CC intermediate Coverslip without BB (barcode blowoff)
42. Apply Cell Conditioner #1
43. Apply CC intermediate Coverslip without BB (barcode blowoff)
44. Apply Cell Conditioner #1
45. Apply CC intermediate Coverslip without BB (barcode blowoff)
46. Apply Cell Conditioner #1
47. Apply CC intermediate Coverslip without BB (barcode blowoff)
48. [STANDARD]
49. Apply Cell Conditioner #1
50. Apply CC intermediate Coverslip without BB (barcode blowoff)
51. Apply Cell Conditioner #1 (brief)
52. Apply CC intermediate Coverslip without BB (barcode blowoff)
53. Apply Cell Conditioner #1
54. Apply CC intermediate Coverslip without BB (barcode blowoff)
55. Deactivate heater from slides
56. Incubate for 8 minutes
57. Wash slides with reaction buffer
58. Adjust volume of slides with reaction buffer
59. Apply coverslip
60. Wash slides with reaction buffer
61. Adjust volume of slides with reaction buffer
62. Apply coverslip
63. Pause (Pause Zone)
64. Heat slides until 36º C
65. Wash slides with reaction buffer
66. Adjust volume of slides with reaction buffer
67. Apply one drop of UV INHIBITOR, apply coverslip and incubate for 4 minutes
68. Wash slides with reaction buffer
69. Adjust volume of slides with reaction buffer
70. Apply coverslip
71. Heat slides to 42ºCand incubate for 4 minutes
72. Wash slides with reaction buffer
73. Adjust volume of slides with reaction buffer
74. Apply one drop of [PREP KIT 35] (Antibody), apply coverslip and incubate (40 min)
75. Wash slides with reaction buffer
76. Adjust volume of slides with reaction buffer
77. Apply coverslip
78. Heat slides until 36º C
79. Wash slides with reaction buffer
80. Apply 100µl + VA Reaction Buffer
81. Apply coverslip
82. Wash slides with reaction buffer
83. Adjust volume of slides with reaction buffer
84. Apply coverslip
85. Wash slides with reaction buffer
86. Adjust volume of slides with reaction buffer
87. Apply coverslip
88. Wash slides with reaction buffer
89. Adjust volume of slides with reaction buffer
90. Apply one drop of UV DAB and one drop of UV DAB H2O2. Apply coverslip, incubate for 8 minutes.
91. Wash slides with reaction buffer
92. Adjust volume of slides with reaction buffer
93. Apply one drop of UV COPPER. Apply coverslip and incubate for 4 minutes.
94. Wash slides with reaction buffer
95. Apply coverslip
96. Deactivate Slide heater
97. Wash slides with reaction buffer

# S1 Fig. **Script in VBA (Visual Basic for Applications) used in Fiji ImageJ to process H-DAB**. *This is the S1 Fig legend:* In line 31 saveAs(“Tiff”, “<put here the path where the files should be saved>” + name);

# S2 Fig. **Script to analyze DAB images into ROI.** *This is the S2 Fig legend:* A video of the process can be found at <https://www.youtube.com/watch?v=9nLRSquNa5Q>
