# Supplementary figures and images for: BCL6 (B-cell lymphoma 6) expression in adenomyosis, leiomyomas and normal myometrium
Source: PLoS One. 2025 Feb 4;20(2):e0317136. doi: 10.1371/journal.pone.0317136 (PMC11793761; doi:10.1371/journal.pone.0317136)

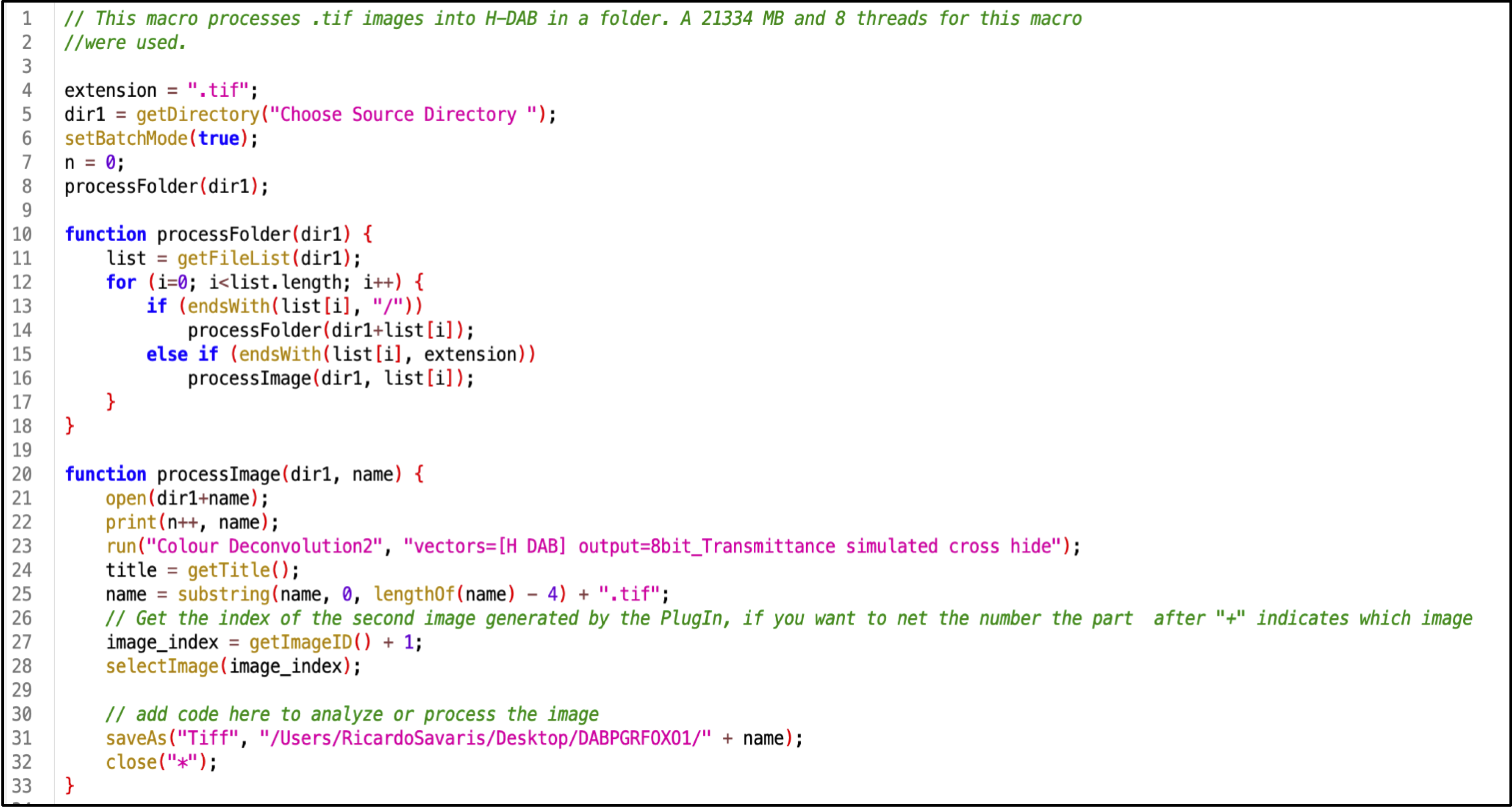

Supplement: S1 Fig — In line 31 saveAs(“Tiff”, “” + name). (TIFF) [file pone.0317136.s002.tiff]

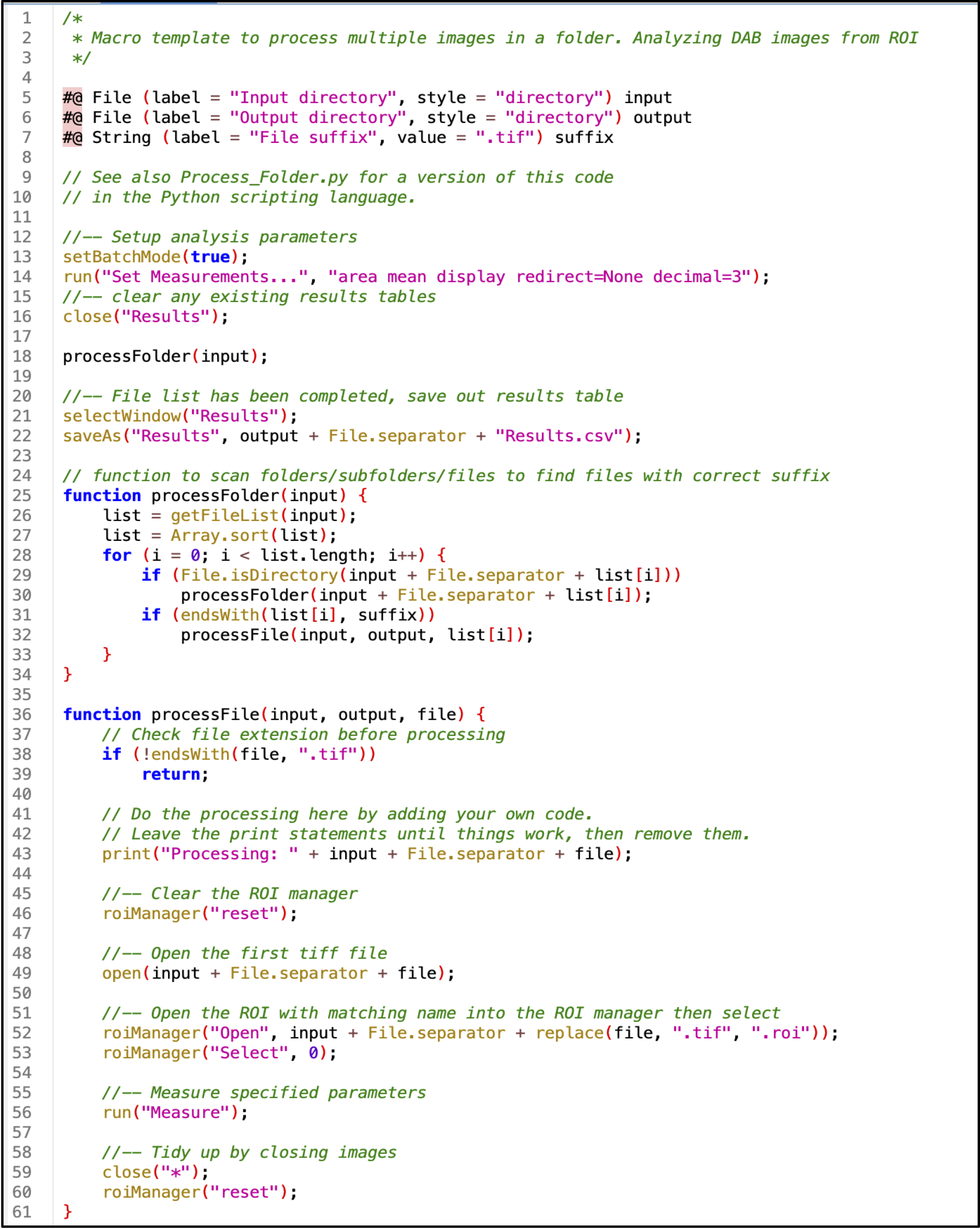

Supplement: S2 Fig — A video of the process can be found at https://www.youtube.com/watch?v=9nLRSquNa5Q. (TIFF) [file pone.0317136.s003.tiff]
